# Korean JOint RegistrY for ALZheimer's Treatment and Diagnostics (JOY‐ALZ): Establishing a Nationwide RWD Registry for Alzheimer's Disease in Korea

**DOI:** 10.1002/alz70858_106260

**Published:** 2025-12-26

**Authors:** Geon Ha Kim, Danbee Kang, Sung Hoon Kang, Jae Seung Kim, Young Chul Youn, Jae‐Sung Lim, So Young Moon, Won‐Jin Moon, Young Ho Park, YongSoo Shim, Dong Won Yang, Hanna Cho, Hojin Choi, Jung‐Min Pyun, Kee Hyung Park, Seong Hye Choi

**Affiliations:** ^1^ Neurology, Ewha Womans University Mokdong Hospital, Ewha Womans University College of Medicine, Seoul, Korea, Republic of (South); ^2^ Samsung Medical Center, Seoul, Korea, Republic of (South); ^3^ SAIHST, Sungkyunkwan University, Seoul, Korea, Republic of (South); ^4^ Korea University Guro Hospital, College of Medicine Korea University, Seoul, Korea, Republic of (South); ^5^ Department of Nuclear Medicine, Asan Medical Center, University of Ulsan College of Medicine, Seoul, Korea, Republic of (South); ^6^ Chung‐Ang University Hospital, Seoul, Korea, Republic of (South); ^7^ Asan Medical Center, Seoul, Seoul, Korea, Republic of (South); ^8^ Ajou University School of Medicine, Suwon, Gyeonggi‐do, Korea, Republic of (South); ^9^ Konkuk University Hospital, Seoul, NA, Korea, Republic of (South); ^10^ Seoul National University Bundang Hospital, Seongnam, Korea, Republic of (South); ^11^ The Catholic University of Korea, St. Vincent's Hospital, Suwon, Gyeonggi‐do, Korea, Republic of (South); ^12^ Seoul St. Mary's Hospital, College of Medicine, The Catholic University of Korea, Seoul, Korea, Republic of (South); ^13^ Gangnam Severance Hospital, Yonsei University College of Medicine, Seoul, Korea, Republic of (South); ^14^ Hanyang University Guri Hospital, Guri, gyeonggi‐do, Korea, Republic of (South); ^15^ Soonchunhyang University Seoul Hospital, Seoul, Korea, Republic of (South); ^16^ Gachon University Gil Medical Center, Gachon University College of Medicine, Incheon, Korea, Republic of (South); ^17^ Inha University School of Medicine, Incheon, Korea, Republic of (South); ^18^ Inha University Hospital, Incheon, Korea, Republic of (South)

## Abstract

Alzheimer's disease (AD) is undergoing a transformation with disease‐modifying therapies (DMTs) and advanced biomarkers, shifting from symptomatic management to targeted interventions. However, high costs, potential side effects, and limited awareness hinder real‐world integration, particularly in underrepresented East Asian populations like Koreans. Preliminary data suggest East Asians may experience lower rates of Amyloid‐Related Imaging Abnormalities (ARIA), emphasizing the need for region‐specific treatment guidelines.

To address this, the Korean Dementia Association (KDA) launched JOY‐ALZ (Korean JOint RegistrY for ALZheimer's Treatment and Diagnostics) in 2024 to establish a real‐world data (RWD) platform for AD therapies in Korea.

Overall objectives of **JOY‐ALZ** are:

• **D**evelop a collaborative network across multiple sites for systemic patient data collection.

• **R**ecord comprehensive patient data on cognitive, functional, and safety outcomes.

• **E**nhance data acquisition by integrating neuroimaging, genetic, and biomarker data

• **A**ssess health outcomes using existing databases for long‐term evaluation.

• **M**aximize data sharing with researchers and stakeholders.

To achieve this, JOY‐ALZ employs the CURE Framework, which encompasses four specific aims:

• Creating the RWD platform infrastructure

• Uncovering outcomes: collecting data to evaluate long term safety and clinical outcomes

• Reinforcing collaborations: co‐enrollment with affiliated studies

• Enabling global sharing: data sharing and linking

Following Korean regulatory approval of lecanemab in May 2024, JOY‐ALZ published Appropriate Use Recommendations (AUR) in October 2024 and hosted educational symposia. IRB submissions were initiated in December 2024 across 41 hospitals, with approval expected by June 2025 to enable comprehensive real‐world data (RWD) collection, including clinical data, neuroimaging, and biosamples.

In the long term, JOY‐ALZ will complement global platforms like ALZ‐NET, informing Korean‐specific treatment guidelines, promoting equitable access to AD therapies, and fostering international collaboration by contributing high‐quality RWD to the global research community. **JOYALZ will play a key role in advancing AD research by leveraging RWD collected through Korea's dementia clinic network**.

